# *N*-(2-{[5-Bromo-2-(piperidin-1-yl)pyrimidin-4-yl]sulfan­yl}-4-meth­oxy­phen­yl)-4-methyl­benzene­sulfonamide

**DOI:** 10.1107/S1600536812037257

**Published:** 2012-09-01

**Authors:** Mohan Kumar, L. Mallesha, M. A. Sridhar, Kamini Kapoor, Vivek K. Gupta, Rajni Kant

**Affiliations:** a Department of Studies in Physics, Manasagangotri, University of Mysore, Mysore 570 006, India; b PG Department of Studies in Chemistry, JSS College of Arts, Commerce and Science, Ooty Road, Mysore 570 025, India; c X-ray Crystallography Laboratory, Post-Graduate Department of Physics & Electronics, University of Jammu, Jammu Tawi 180 006, India

## Abstract

In the title compound, C_23_H_25_BrN_4_O_3_S_2_, the benzene rings bridged by the sulfonamide group are tilted relative to each other by 69.7 (1)° and the dihedral angle between the sulfur-bridged pyrimidine and benzene rings is 70.4 (1)°. The mol­ecular conformation is stabilized by a weak intra­molecular π–π stacking inter­action between the pyrimidine and the 4-methyl benzene rings [centroid–centroid distance = 3.633 (2) Å]. The piperidine ring adopts a chair conformation. In the crystal, mol­ecules are linked into inversion dimers by pairs of N—H⋯O hydrogen bonds.

## Related literature

For a related structure and background to sulfonamides, see: Kant *et al.* (2012 ▶).
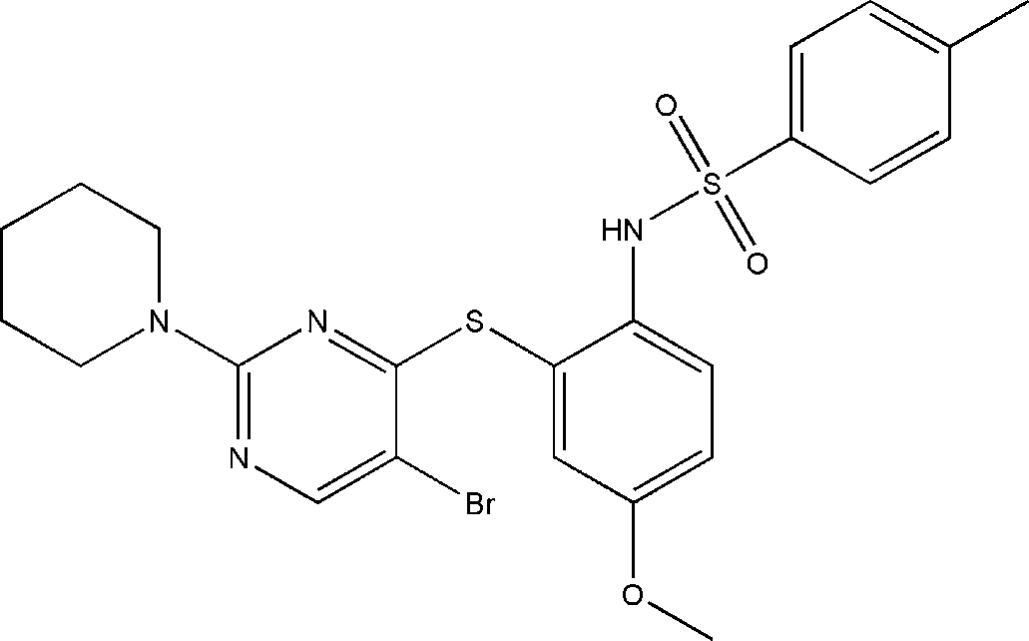


## Experimental

### 

#### Crystal data

C_23_H_25_BrN_4_O_3_S_2_
*M* *_r_* = 549.50
Triclinic, 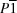

*a* = 9.8318 (3) Å
*b* = 10.3822 (3) Å
*c* = 13.4393 (4) Å
α = 96.654 (3)°
β = 103.085 (3)°
γ = 107.714 (3)°
*V* = 1247.36 (6) Å^3^
*Z* = 2
Mo *K*α radiation
μ = 1.85 mm^−1^
*T* = 293 K
0.30 × 0.20 × 0.20 mm

#### Data collection

Oxford Diffraction Xcalibur CCD, Sapphire3 diffractometer
Absorption correction: multi-scan (*CrysAlis PRO*; Oxford Diffraction, 2010 ▶) *T*_min_ = 0.764, *T*_max_ = 1.000
41580 measured reflections
4385 independent reflections
3645 reflections with *I* > 2σ(*I*)
*R*_int_ = 0.038

#### Refinement

*R*[*F* ^2^ > 2σ(*F* ^2^)] = 0.037
*wR*(*F* ^2^) = 0.093
*S* = 1.03
4385 reflections
300 parameters
H-atom parameters constrained
Δρ_max_ = 0.66 e Å^−3^
Δρ_min_ = −0.52 e Å^−3^

### 

Data collection: *CrysAlis PRO* (Oxford Diffraction, 2010 ▶); cell refinement: *CrysAlis PRO*; data reduction: *CrysAlis PRO*; program(s) used to solve structure: *SHELXS97* (Sheldrick, 2008 ▶); program(s) used to refine structure: *SHELXL97* (Sheldrick, 2008 ▶); molecular graphics: *ORTEP-3* (Farrugia, 1997 ▶); software used to prepare material for publication: *PLATON* (Spek, 2009 ▶).

## Supplementary Material

Crystal structure: contains datablock(s) I, global. DOI: 10.1107/S1600536812037257/hb6947sup1.cif

Structure factors: contains datablock(s) I. DOI: 10.1107/S1600536812037257/hb6947Isup2.hkl

Supplementary material file. DOI: 10.1107/S1600536812037257/hb6947Isup3.cml

Additional supplementary materials:  crystallographic information; 3D view; checkCIF report

## Figures and Tables

**Table 1:** Hydrogen-bond geometry (Å, °)

*D*—H⋯*A*	*D*—H	H⋯*A*	*D*⋯*A*	*D*—H⋯*A*
N8—H8⋯O2^i^	0.86	2.22	2.955 (4)	143

Symmetry code: (i) 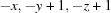.

**Fig. 1.**
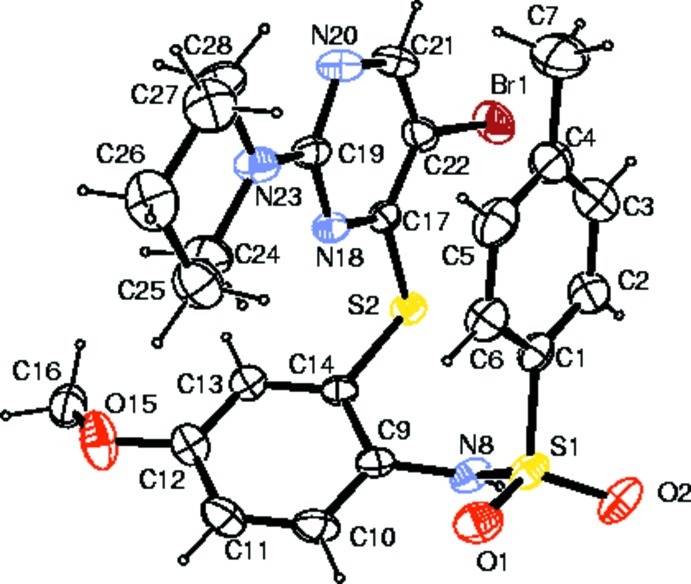
The molecular structure of (I) with displacement ellipsoids drawn at the 40% probability level.

**Fig. 2.**
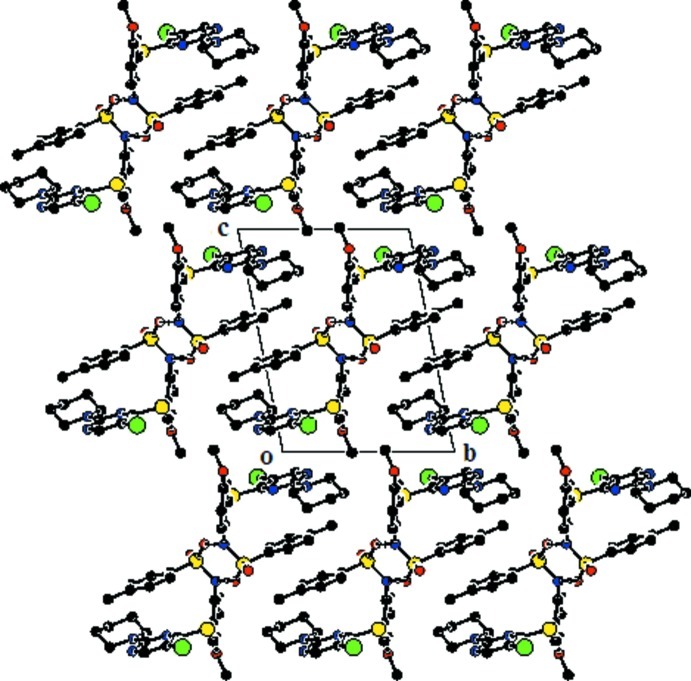
A unit-cell packing view of the title compound down the a axis, showing intermolecular interactions. For clarity, hydrogen atoms which are not involved in hydrogen bonding have been omitted.

## supplementary crystallographic information

### Comment

As part of our ongoing studies of sulfonamides (Kant *et al.*, 2012), we now report the structure of the title compound, (I), (Fig. 1).

The piperidine ring is exhibiting a chair conformation. The two benzene rings (C1—C6/C9—C14) are tilted relative to each other by 69.7 (1)° and the dihedral angle between the sulfur bridged pyrimidine and benzene rings is 70.4 (1)°. The molecular conformation is stabilized by a weak intramolecular stacking interaction between the pyrimidine and the 4 -methyl benzene rings [centroid–centroid distance = 3.633 (2) Å, interplanar spacing = 3.494 Å, and centroid shift = 1.00 Å]. In the crystal, molecules are linked into inversion dimers by pairs of N8—H8···O2 hydrogen bonds (Fig.2).

### Experimental

The reaction of *N*-[2-(5-bromo-2-piperidin-1-yl-pyrimidin-4-ylsulfanyl)-4-methoxy -phenyl]-4-methyl-benzenesulfonamide (5.01 g, 0.01 mol) and piperidine (0.86 g, 0.01 mol) were carried out in the presence of triethylamine and the reaction mixture was allowed to stir at room temperature for 6–7 h in dry dichloromethane. The progress of the reaction was monitored by TLC. Upon completion, the solvent was removed under reduced pressure and residue was extracted with ethyl acetate. The compound was purified by successive recrystallization from methanol (yield 83%, m. p. 483–485 K) to yield light brown blocks of (I).

### Refinement

All H atoms were positioned geometrically and were treated as riding on their parent C atoms, with C—H distances of 0.93–0.97 Å and with *U*_iso_(H) = 1.2*U*_eq_(C) or 1.5*U*_eq_(methyl C).

### Crystal data

**Table:**

C_23_H_25_BrN_4_O_3_S_2_	*Z* = 2
*M**_r_* = 549.50	*F*(000) = 564
Triclinic, *P*1	*D*_x_ = 1.463 Mg m^−^^3^
Hall symbol: -P 1	Mo *K*α radiation, λ = 0.71073 Å
*a* = 9.8318 (3) Å	Cell parameters from 16775 reflections
*b* = 10.3822 (3) Å	θ = 3.4–29.1°
*c* = 13.4393 (4) Å	µ = 1.85 mm^−^^1^
α = 96.654 (3)°	*T* = 293 K
β = 103.085 (3)°	Block, light-brown
γ = 107.714 (3)°	0.30 × 0.20 × 0.20 mm
*V* = 1247.36 (6) Å^3^	

### Data collection

**Table:**

Oxford Diffraction Xcalibur CCD, Sapphire3 diffractometer	4385 independent reflections
Radiation source: fine-focus sealed tube	3645 reflections with *I* > 2σ(*I*)
Graphite monochromator	*R*_int_ = 0.038
Detector resolution: 16.1049 pixels mm^-1^	θ_max_ = 25.0°, θ_min_ = 3.4°
ω scans	*h* = −11→11
Absorption correction: multi-scan (*CrysAlis PRO*; Oxford Diffraction, 2010)	*k* = −12→12
*T*_min_ = 0.764, *T*_max_ = 1.000	*l* = −15→15
41580 measured reflections	

### Refinement

**Table:**

Refinement on *F*^2^	Primary atom site location: structure-invariant direct methods
Least-squares matrix: full	Secondary atom site location: difference Fourier map
*R*[*F*^2^ > 2σ(*F*^2^)] = 0.037	Hydrogen site location: inferred from neighbouring sites
*wR*(*F*^2^) = 0.093	H-atom parameters constrained
*S* = 1.03	*w* = 1/[σ^2^(*F*_o_^2^) + (0.0396*P*)^2^ + 0.9744*P*] where *P* = (*F*_o_^2^ + 2*F*_c_^2^)/3
4385 reflections	(Δ/σ)_max_ = 0.001
300 parameters	Δρ_max_ = 0.66 e Å^−^^3^
0 restraints	Δρ_min_ = −0.52 e Å^−^^3^

### Special details

**Table:**

Experimental. *CrysAlis PRO*, Oxford Diffraction Ltd., Version 1.171.34.40 (release 27–08-2010 CrysAlis171. NET) (compiled Aug 27 2010,11:50:40) Empirical absorption correction using spherical harmonics, implemented in SCALE3 ABSPACK scaling algorithm.
Geometry. All e.s.d.'s (except the e.s.d. in the dihedral angle between two l.s. planes) are estimated using the full covariance matrix. The cell e.s.d.'s are taken into account individually in the estimation of e.s.d.'s in distances, angles and torsion angles; correlations between e.s.d.'s in cell parameters are only used when they are defined by crystal symmetry. An approximate (isotropic) treatment of cell e.s.d.'s is used for estimating e.s.d.'s involving l.s. planes.
Refinement. Refinement of *F*^2^ against ALL reflections. The weighted *R*-factor *wR* and goodness of fit *S* are based on *F*^2^, conventional *R*-factors *R* are based on *F*, with *F* set to zero for negative *F*^2^. The threshold expression of *F*^2^ > σ(*F*^2^) is used only for calculating *R*-factors(gt) *etc*. and is not relevant to the choice of reflections for refinement. *R*-factors based on *F*^2^ are statistically about twice as large as those based on *F*, and *R*- factors based on ALL data will be even larger.

### Fractional atomic coordinates and isotropic or equivalent isotropic displacement parameters (Å^2^)

**Table:**

	*x*	*y*	*z*	*U*_iso_*/*U*_eq_	
Br1	0.00669 (4)	0.82027 (4)	0.88239 (3)	0.06852 (14)	
S1	0.22673 (8)	0.63699 (8)	0.49289 (5)	0.04950 (19)	
S2	0.20227 (7)	0.65245 (7)	0.79943 (5)	0.04366 (17)	
O1	0.3610 (3)	0.6577 (2)	0.46369 (16)	0.0639 (6)	
O2	0.0875 (3)	0.5665 (2)	0.41649 (15)	0.0656 (6)	
C1	0.2248 (3)	0.7998 (3)	0.5459 (2)	0.0449 (6)	
C2	0.0995 (4)	0.8109 (3)	0.5695 (3)	0.0582 (8)	
H2	0.0164	0.7326	0.5593	0.070*	
C3	0.0980 (4)	0.9391 (4)	0.6086 (3)	0.0696 (9)	
H3	0.0134	0.9463	0.6252	0.084*	
C4	0.2194 (4)	1.0571 (3)	0.6237 (3)	0.0642 (9)	
C5	0.3433 (4)	1.0432 (3)	0.6002 (3)	0.0672 (9)	
H5	0.4261	1.1216	0.6100	0.081*	
C6	0.3486 (3)	0.9157 (3)	0.5622 (2)	0.0574 (8)	
H6	0.4344	0.9082	0.5479	0.069*	
C7	0.2160 (6)	1.1970 (4)	0.6657 (4)	0.1016 (15)	
H7A	0.3102	1.2506	0.7142	0.152*	
H7B	0.1391	1.1858	0.7005	0.152*	
H7C	0.1965	1.2435	0.6092	0.152*	
N8	0.2273 (3)	0.5458 (2)	0.58395 (17)	0.0485 (6)	
H8	0.1476	0.4793	0.5805	0.058*	
C9	0.3552 (3)	0.5726 (3)	0.6691 (2)	0.0428 (6)	
C10	0.4798 (4)	0.5487 (3)	0.6516 (2)	0.0599 (8)	
H10	0.4800	0.5182	0.5838	0.072*	
C11	0.6028 (4)	0.5690 (4)	0.7322 (3)	0.0629 (8)	
H11	0.6868	0.5558	0.7185	0.076*	
C12	0.6023 (3)	0.6090 (3)	0.8336 (2)	0.0527 (7)	
C13	0.4792 (3)	0.6322 (3)	0.8528 (2)	0.0451 (6)	
H13	0.4783	0.6592	0.9210	0.054*	
C14	0.3561 (3)	0.6157 (2)	0.7709 (2)	0.0391 (6)	
O15	0.7290 (2)	0.6271 (3)	0.9099 (2)	0.0810 (8)	
C16	0.7150 (4)	0.5974 (4)	1.0061 (3)	0.0660 (9)	
H16A	0.6837	0.6649	1.0407	0.099*	
H16B	0.8090	0.5995	1.0480	0.099*	
H16C	0.6425	0.5074	0.9960	0.099*	
C17	0.2812 (3)	0.8343 (3)	0.83612 (18)	0.0391 (6)	
N18	0.4152 (2)	0.8963 (2)	0.82825 (16)	0.0399 (5)	
C19	0.4729 (3)	1.0343 (3)	0.8579 (2)	0.0444 (6)	
N20	0.4065 (3)	1.1143 (2)	0.8998 (2)	0.0560 (6)	
C21	0.2713 (4)	1.0476 (3)	0.9059 (2)	0.0584 (8)	
H21	0.2212	1.0990	0.9337	0.070*	
C22	0.2008 (3)	0.9085 (3)	0.8739 (2)	0.0480 (7)	
N23	0.6066 (3)	1.0965 (2)	0.8425 (2)	0.0548 (6)	
C24	0.6954 (4)	1.0140 (3)	0.8157 (3)	0.0648 (9)	
H24A	0.6300	0.9209	0.7819	0.078*	
H24B	0.7626	1.0088	0.8789	0.078*	
C25	0.7831 (4)	1.0749 (4)	0.7447 (3)	0.0768 (10)	
H25A	0.7158	1.0658	0.6771	0.092*	
H25B	0.8488	1.0242	0.7350	0.092*	
C26	0.8740 (4)	1.2256 (4)	0.7879 (3)	0.0828 (11)	
H26A	0.9498	1.2343	0.8512	0.099*	
H26B	0.9233	1.2638	0.7377	0.099*	
C27	0.7764 (4)	1.3046 (4)	0.8112 (3)	0.0788 (11)	
H27A	0.8377	1.3992	0.8440	0.095*	
H27B	0.7097	1.3061	0.7463	0.095*	
C28	0.6865 (4)	1.2435 (3)	0.8813 (3)	0.0685 (9)	
H28A	0.7519	1.2578	0.9506	0.082*	
H28B	0.6160	1.2903	0.8865	0.082*	

### Atomic displacement parameters (Å^2^)

**Table:**

	*U*^11^	*U*^22^	*U*^33^	*U*^12^	*U*^13^	*U*^23^
Br1	0.0502 (2)	0.0873 (3)	0.0799 (3)	0.03164 (18)	0.02998 (17)	0.01633 (19)
S1	0.0587 (4)	0.0511 (4)	0.0322 (3)	0.0106 (3)	0.0140 (3)	0.0029 (3)
S2	0.0408 (4)	0.0443 (4)	0.0436 (4)	0.0102 (3)	0.0162 (3)	0.0030 (3)
O1	0.0750 (15)	0.0739 (15)	0.0493 (12)	0.0249 (12)	0.0317 (11)	0.0101 (11)
O2	0.0735 (15)	0.0658 (14)	0.0344 (10)	0.0018 (11)	0.0058 (10)	−0.0003 (10)
C1	0.0497 (16)	0.0481 (16)	0.0329 (13)	0.0118 (13)	0.0089 (12)	0.0108 (12)
C2	0.0544 (18)	0.0514 (18)	0.066 (2)	0.0141 (14)	0.0163 (15)	0.0143 (15)
C3	0.070 (2)	0.072 (2)	0.079 (2)	0.0362 (19)	0.0254 (19)	0.0203 (19)
C4	0.081 (2)	0.0513 (19)	0.058 (2)	0.0264 (18)	0.0082 (17)	0.0154 (15)
C5	0.071 (2)	0.0516 (19)	0.061 (2)	0.0040 (16)	0.0074 (17)	0.0133 (16)
C6	0.0541 (18)	0.0562 (19)	0.0553 (18)	0.0088 (15)	0.0157 (14)	0.0115 (15)
C7	0.137 (4)	0.063 (2)	0.107 (3)	0.050 (3)	0.019 (3)	0.012 (2)
N8	0.0601 (15)	0.0382 (12)	0.0391 (12)	0.0074 (11)	0.0136 (11)	0.0029 (10)
C9	0.0565 (16)	0.0323 (13)	0.0421 (15)	0.0160 (12)	0.0181 (13)	0.0060 (11)
C10	0.082 (2)	0.065 (2)	0.0509 (18)	0.0386 (18)	0.0351 (17)	0.0115 (15)
C11	0.065 (2)	0.076 (2)	0.071 (2)	0.0401 (18)	0.0359 (18)	0.0246 (18)
C12	0.0499 (17)	0.0580 (18)	0.0585 (18)	0.0205 (14)	0.0216 (14)	0.0241 (15)
C13	0.0482 (15)	0.0457 (15)	0.0420 (15)	0.0128 (12)	0.0170 (12)	0.0111 (12)
C14	0.0446 (14)	0.0304 (13)	0.0436 (14)	0.0107 (11)	0.0181 (12)	0.0065 (11)
O15	0.0464 (13)	0.125 (2)	0.0810 (17)	0.0292 (13)	0.0216 (12)	0.0489 (16)
C16	0.062 (2)	0.065 (2)	0.062 (2)	0.0191 (17)	0.0039 (16)	0.0112 (17)
C17	0.0440 (15)	0.0459 (15)	0.0286 (12)	0.0183 (12)	0.0095 (11)	0.0053 (11)
N18	0.0460 (12)	0.0388 (12)	0.0372 (11)	0.0153 (10)	0.0164 (10)	0.0046 (9)
C19	0.0560 (17)	0.0430 (15)	0.0368 (14)	0.0187 (13)	0.0165 (12)	0.0052 (12)
N20	0.0729 (17)	0.0441 (14)	0.0582 (15)	0.0233 (13)	0.0301 (13)	0.0045 (11)
C21	0.074 (2)	0.0546 (19)	0.0634 (19)	0.0356 (17)	0.0342 (17)	0.0089 (15)
C22	0.0498 (16)	0.0595 (18)	0.0442 (15)	0.0268 (14)	0.0198 (13)	0.0107 (13)
N23	0.0581 (15)	0.0410 (13)	0.0651 (16)	0.0119 (11)	0.0273 (13)	0.0036 (11)
C24	0.0604 (19)	0.0529 (19)	0.085 (2)	0.0163 (15)	0.0339 (18)	0.0089 (17)
C25	0.069 (2)	0.091 (3)	0.084 (3)	0.033 (2)	0.037 (2)	0.021 (2)
C26	0.065 (2)	0.087 (3)	0.101 (3)	0.016 (2)	0.036 (2)	0.039 (2)
C27	0.080 (2)	0.062 (2)	0.094 (3)	0.0160 (19)	0.027 (2)	0.029 (2)
C28	0.076 (2)	0.0458 (18)	0.073 (2)	0.0069 (16)	0.0225 (18)	0.0026 (16)

### Geometric parameters (Å, º)

**Table:**

Br1—C22	1.885 (3)	C13—C14	1.390 (4)
S1—O1	1.423 (2)	C13—H13	0.9300
S1—O2	1.429 (2)	O15—C16	1.390 (4)
S1—N8	1.632 (2)	C16—H16A	0.9600
S1—C1	1.764 (3)	C16—H16B	0.9600
S2—C17	1.769 (3)	C16—H16C	0.9600
S2—C14	1.778 (3)	C17—N18	1.313 (3)
C1—C2	1.374 (4)	C17—C22	1.393 (4)
C1—C6	1.380 (4)	N18—C19	1.343 (3)
C2—C3	1.379 (5)	C19—N20	1.350 (3)
C2—H2	0.9300	C19—N23	1.351 (4)
C3—C4	1.382 (5)	N20—C21	1.325 (4)
C3—H3	0.9300	C21—C22	1.365 (4)
C4—C5	1.370 (5)	C21—H21	0.9300
C4—C7	1.509 (5)	N23—C28	1.456 (4)
C5—C6	1.383 (5)	N23—C24	1.467 (4)
C5—H5	0.9300	C24—C25	1.494 (5)
C6—H6	0.9300	C24—H24A	0.9700
C7—H7A	0.9600	C24—H24B	0.9700
C7—H7B	0.9600	C25—C26	1.513 (5)
C7—H7C	0.9600	C25—H25A	0.9700
N8—C9	1.425 (4)	C25—H25B	0.9700
N8—H8	0.8600	C26—C27	1.499 (5)
C9—C10	1.385 (4)	C26—H26A	0.9700
C9—C14	1.386 (4)	C26—H26B	0.9700
C10—C11	1.371 (5)	C27—C28	1.498 (5)
C10—H10	0.9300	C27—H27A	0.9700
C11—C12	1.380 (4)	C27—H27B	0.9700
C11—H11	0.9300	C28—H28A	0.9700
C12—O15	1.369 (4)	C28—H28B	0.9700
C12—C13	1.376 (4)		
			
O1—S1—O2	119.46 (13)	O15—C16—H16A	109.5
O1—S1—N8	108.46 (14)	O15—C16—H16B	109.5
O2—S1—N8	104.72 (13)	H16A—C16—H16B	109.5
O1—S1—C1	107.97 (14)	O15—C16—H16C	109.5
O2—S1—C1	107.66 (14)	H16A—C16—H16C	109.5
N8—S1—C1	108.12 (12)	H16B—C16—H16C	109.5
C17—S2—C14	99.34 (12)	N18—C17—C22	121.4 (3)
C2—C1—C6	120.3 (3)	N18—C17—S2	119.23 (19)
C2—C1—S1	119.8 (2)	C22—C17—S2	119.4 (2)
C6—C1—S1	119.9 (2)	C17—N18—C19	117.9 (2)
C1—C2—C3	119.4 (3)	N18—C19—N20	125.1 (3)
C1—C2—H2	120.3	N18—C19—N23	116.7 (2)
C3—C2—H2	120.3	N20—C19—N23	118.2 (3)
C2—C3—C4	121.5 (3)	C21—N20—C19	115.0 (3)
C2—C3—H3	119.3	N20—C21—C22	124.3 (3)
C4—C3—H3	119.3	N20—C21—H21	117.8
C5—C4—C3	118.0 (3)	C22—C21—H21	117.8
C5—C4—C7	121.0 (4)	C21—C22—C17	116.3 (3)
C3—C4—C7	121.0 (4)	C21—C22—Br1	122.2 (2)
C4—C5—C6	121.7 (3)	C17—C22—Br1	121.5 (2)
C4—C5—H5	119.1	C19—N23—C28	121.4 (2)
C6—C5—H5	119.1	C19—N23—C24	120.1 (2)
C1—C6—C5	119.1 (3)	C28—N23—C24	115.9 (3)
C1—C6—H6	120.4	N23—C24—C25	111.5 (3)
C5—C6—H6	120.4	N23—C24—H24A	109.3
C4—C7—H7A	109.5	C25—C24—H24A	109.3
C4—C7—H7B	109.5	N23—C24—H24B	109.3
H7A—C7—H7B	109.5	C25—C24—H24B	109.3
C4—C7—H7C	109.5	H24A—C24—H24B	108.0
H7A—C7—H7C	109.5	C24—C25—C26	111.6 (3)
H7B—C7—H7C	109.5	C24—C25—H25A	109.3
C9—N8—S1	122.28 (19)	C26—C25—H25A	109.3
C9—N8—H8	118.9	C24—C25—H25B	109.3
S1—N8—H8	118.9	C26—C25—H25B	109.3
C10—C9—C14	118.4 (3)	H25A—C25—H25B	108.0
C10—C9—N8	120.0 (2)	C27—C26—C25	110.4 (3)
C14—C9—N8	121.5 (2)	C27—C26—H26A	109.6
C11—C10—C9	121.4 (3)	C25—C26—H26A	109.6
C11—C10—H10	119.3	C27—C26—H26B	109.6
C9—C10—H10	119.3	C25—C26—H26B	109.6
C10—C11—C12	120.0 (3)	H26A—C26—H26B	108.1
C10—C11—H11	120.0	C28—C27—C26	112.5 (3)
C12—C11—H11	120.0	C28—C27—H27A	109.1
O15—C12—C13	123.8 (3)	C26—C27—H27A	109.1
O15—C12—C11	116.7 (3)	C28—C27—H27B	109.1
C13—C12—C11	119.5 (3)	C26—C27—H27B	109.1
C12—C13—C14	120.4 (3)	H27A—C27—H27B	107.8
C12—C13—H13	119.8	N23—C28—C27	111.5 (3)
C14—C13—H13	119.8	N23—C28—H28A	109.3
C9—C14—C13	120.1 (2)	C27—C28—H28A	109.3
C9—C14—S2	121.1 (2)	N23—C28—H28B	109.3
C13—C14—S2	118.7 (2)	C27—C28—H28B	109.3
C12—O15—C16	118.1 (2)	H28A—C28—H28B	108.0
			
O1—S1—C1—C2	172.6 (2)	C12—C13—C14—S2	−178.0 (2)
O2—S1—C1—C2	42.4 (3)	C17—S2—C14—C9	−108.1 (2)
N8—S1—C1—C2	−70.2 (3)	C17—S2—C14—C13	71.3 (2)
O1—S1—C1—C6	−6.6 (3)	C13—C12—O15—C16	34.4 (5)
O2—S1—C1—C6	−136.8 (2)	C11—C12—O15—C16	−147.6 (3)
N8—S1—C1—C6	110.5 (2)	C14—S2—C17—N18	4.4 (2)
C6—C1—C2—C3	0.7 (5)	C14—S2—C17—C22	−174.8 (2)
S1—C1—C2—C3	−178.5 (2)	C22—C17—N18—C19	0.0 (4)
C1—C2—C3—C4	0.6 (5)	S2—C17—N18—C19	−179.23 (19)
C2—C3—C4—C5	−1.0 (5)	C17—N18—C19—N20	3.2 (4)
C2—C3—C4—C7	179.5 (3)	C17—N18—C19—N23	−175.7 (2)
C3—C4—C5—C6	0.1 (5)	N18—C19—N20—C21	−3.4 (4)
C7—C4—C5—C6	179.6 (3)	N23—C19—N20—C21	175.5 (3)
C2—C1—C6—C5	−1.6 (4)	C19—N20—C21—C22	0.5 (5)
S1—C1—C6—C5	177.6 (2)	N20—C21—C22—C17	2.2 (5)
C4—C5—C6—C1	1.2 (5)	N20—C21—C22—Br1	−178.8 (2)
O1—S1—N8—C9	43.7 (2)	N18—C17—C22—C21	−2.5 (4)
O2—S1—N8—C9	172.3 (2)	S2—C17—C22—C21	176.7 (2)
C1—S1—N8—C9	−73.1 (2)	N18—C17—C22—Br1	178.55 (19)
S1—N8—C9—C10	−67.8 (3)	S2—C17—C22—Br1	−2.3 (3)
S1—N8—C9—C14	115.5 (3)	N18—C19—N23—C28	−173.6 (3)
C14—C9—C10—C11	−1.1 (4)	N20—C19—N23—C28	7.4 (4)
N8—C9—C10—C11	−177.8 (3)	N18—C19—N23—C24	−12.3 (4)
C9—C10—C11—C12	2.5 (5)	N20—C19—N23—C24	168.7 (3)
C10—C11—C12—O15	179.9 (3)	C19—N23—C24—C25	146.5 (3)
C10—C11—C12—C13	−1.9 (5)	C28—N23—C24—C25	−51.2 (4)
O15—C12—C13—C14	178.0 (3)	N23—C24—C25—C26	52.4 (4)
C11—C12—C13—C14	0.0 (4)	C24—C25—C26—C27	−54.7 (4)
C10—C9—C14—C13	−0.9 (4)	C25—C26—C27—C28	54.2 (5)
N8—C9—C14—C13	175.8 (2)	C19—N23—C28—C27	−147.7 (3)
C10—C9—C14—S2	178.5 (2)	C24—N23—C28—C27	50.3 (4)
N8—C9—C14—S2	−4.8 (3)	C26—C27—C28—N23	−51.4 (5)
C12—C13—C14—C9	1.4 (4)		

### Hydrogen-bond geometry (Å, º)

**Table:**

*D*—H···*A*	*D*—H	H···*A*	*D*···*A*	*D*—H···*A*
N8—H8···O2^i^	0.86	2.22	2.955 (4)	143

Symmetry code: (i) −*x*, −*y*+1, −*z*+1.
